# Supplementary figures and images for: Using prior information from humans to prioritize genes and gene-associated variants for complex traits in livestock
Source: PLoS Genet. 2020 Sep 14;16(9):e1008780. doi: 10.1371/journal.pgen.1008780 (PMC7514049; doi:10.1371/journal.pgen.1008780)

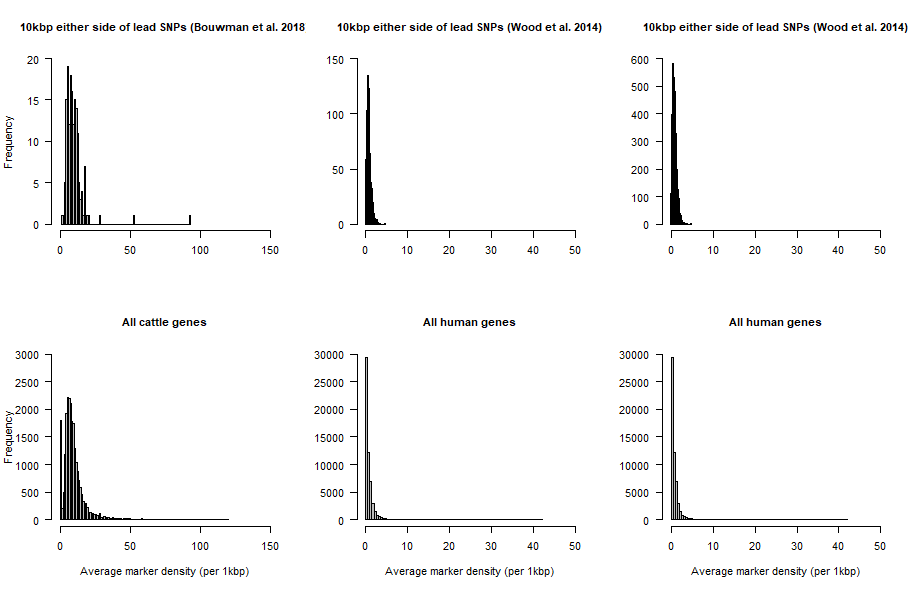

Supplement: S1 Fig — Average marker density were computed as total number of markers in a window or gene divided by the length (in kbp) of the window or gene. (TIF) [file pgen.1008780.s001.tif]
