# Supplementary material for: Using prior information from humans to prioritize genes and gene-associated variants for complex traits in livestock
Source: PLoS Genet. 2020 Sep 14;16(9):e1008780. doi: 10.1371/journal.pgen.1008780 (PMC7514049; doi:10.1371/journal.pgen.1008780)
Supplement: S1 Table — *Cattle genes with 1 to 1 orthologs in humans (after QC); **Cattle genes within 10kb of the 164 lead SNPs from Bouwman et al. [12] that also have orthologs in humans; ***Human height genes prioritized as those that overlap with, or are within 10kkp either side of the 649 lead SNPs for height in Wood et al. [9], that also have orthologs in cattle. The proportion 10/77 is more than can be expected by chance: Fisher’s exact test (odds ratio = 5.5, p-value = 3.7e-05). (DOCX) [file pgen.1008780.s002.docx]

**S1 Table: Contingency table showing the overlap of cattle stature genes from Bouwman *et al*. [12] with human height genes from Wood *et al*. [9].**

|  | In Wood *et al*. [9] | Not in Wood *et al*. [9] | Marginal totals (rows) |
| --- | --- | --- | --- |
| In Bouwman *et al*. [12] | 10 | 67 | **77**** |
| Not In Bouwman *et al*. [12] | 358 | 13,307 | **13,665** |
| Marginal totals (columns) | **368***** | **13,374** | **13,742*** |

*Cattle genes with 1 to 1 orthologs in humans (after QC); **Cattle genes within 10kb of the 164 lead SNPs from Bouwman *et al*. [12] that also have orthologs in humans; ***Human height genes prioritized as those that overlap with, or are within 10kkp either side of the 649 lead SNPs for height in Wood *et al*. [9], that also have orthologs in cattle. The proportion 10/77 is more than can be expected by chance: Fisher’s exact test (odds ratio = 5.5, p-value = 3.7e-05).
