# Supplementary material for: Using prior information from humans to prioritize genes and gene-associated variants for complex traits in livestock
Source: PLoS Genet. 2020 Sep 14;16(9):e1008780. doi: 10.1371/journal.pgen.1008780 (PMC7514049; doi:10.1371/journal.pgen.1008780)
Supplement: S2 Table — (DOCX) [file pgen.1008780.s003.docx]

**S2 Table: Names and coordinates of 10 orthologous that are associated with both human height based on results from Wood *et al*. [9] and cattle stature based on results from Bouwman *et al*. [12].**

| **Gene name** | **Chromosome - Cattle** | **Start (BP) - Cattle** | **Stop (BP) - cattle** | **Chromosome - Human** | **Start (BP) - Human** | **Stop (BP) - Human** |
| --- | --- | --- | --- | --- | --- | --- |
| GATAD2A | 7 | 3737212 | 3838689 | 19 | 19385826 | 19508931 |
| MAN1C1 | 2 | 127888646 | 128008111 | 1 | 25617468 | 25786207 |
| VPS8 | 1 | 82706097 | 82987709 | 3 | 184812143 | 185052614 |
| GNAQ | 8 | 53970972 | 54280697 | 9 | 77716087 | 78031458 |
| DIS3L2 | 2 | 120486066 | 120794558 | 2 | 231961245 | 232344350 |
| ADAM12 | 26 | 45848827 | 46238138 | 10 | 126012381 | 126388455 |
| INSR | 7 | 17280452 | 17403052 | 19 | 7112255 | 7294034 |
| GNA12 | 25 | 41099259 | 41171209 | 7 | 2728112 | 2844324 |
| IGF2BP3 | 4 | 32077891 | 32222388 | 7 | 23310209 | 23470467 |
| DNMT3A | 11 | 74030425 | 74059688 | 2 | 25227855 | 25342590 |
