# Supplementary material for: Using prior information from humans to prioritize genes and gene-associated variants for complex traits in livestock
Source: PLoS Genet. 2020 Sep 14;16(9):e1008780. doi: 10.1371/journal.pgen.1008780 (PMC7514049; doi:10.1371/journal.pgen.1008780)
Supplement: S3 Table — *Cattle genes with 1 to 1 orthologs in humans (after QC); **Cattle genes within 10kbp of the 164 lead SNPs from Bouwman et al. [12] that also have orthologs in humans; ***Human height genes prioritized as those that overlap with, or are within 10kbp either side of the 3,290 lead SNPs for height in Yengo et al. [10], that also have orthologs in cattle. The proportion 30/77 is more than can be expected by chance: Fisher’s exact test (odds ratio = 5.1, p-value = 3.1e-10). (DOCX) [file pgen.1008780.s004.docx]

**S3 Table: Contingency table showing the overlap of cattle stature genes from Bouwman *et al*. [12] with human height genes from Yengo *et al*. [10].**

|  | In Yengo *et al*. [10] | Not In Yengo *et al*. [10] | Marginal totals (rows) |
| --- | --- | --- | --- |
| In Bouwman *et al*. [12] | 30 | 47 | **77**** |
| Not In Bouwman *et al*. [12] | 1,523 | 12,142 | **13,665** |
| Marginal totals (columns) | **1,553***** | **12,189** | **13,742*** |

*Cattle genes with 1 to 1 orthologs in humans (after QC); **Cattle genes within 10kb of the 164 lead SNPs from Bouwman *et al*. [12] that also have orthologs in humans; ***Human height genes prioritized as those that overlap with, or are within 10kbp either side of the 3,290 lead SNPs for height in Yengo *et al*. [10], that also have orthologs in cattle. The proportion 30/77 is more than can be expected by chance: Fisher’s exact test (odds ratio = 5.1, p-value = 3.1e-10).
