# Supplementary material for: Using prior information from humans to prioritize genes and gene-associated variants for complex traits in livestock
Source: PLoS Genet. 2020 Sep 14;16(9):e1008780. doi: 10.1371/journal.pgen.1008780 (PMC7514049; doi:10.1371/journal.pgen.1008780)
Supplement: S4 Table — (DOCX) [file pgen.1008780.s005.docx]

**S4 Table: Names and coordinates of 30 orthologous that are associated with both human height based on results from Yengo *et al*. [10] and cattle stature based on results from Bouwman *et al*. [12].**

| **Gene name** | **Chromosome - Cattle** | **Start (BP) - Cattle** | **Stop (BP) - cattle** | **Chromosome - Human** | **Start (BP) - Human** | **Stop (BP) - Human** |
| --- | --- | --- | --- | --- | --- | --- |
| FBN1 | 10 | 61877808 | 62142171 | 15 | 48408306 | 48645849 |
| MAN1C1 | 2 | 1.28E+08 | 1.28E+08 | 1 | 25617468 | 25786207 |
| AXL | 18 | 50677199 | 50709510 | 19 | 41219203 | 41261766 |
| TBC1D16 | 19 | 53181379 | 53245548 | 17 | 79932343 | 80035848 |
| C13H20orf194 | 13 | 52253039 | 52422570 | 20 | 3249305 | 3407625 |
| ADAMTSL4 | 3 | 20187039 | 20195550 | 1 | 1.51E+08 | 1.51E+08 |
| PLEKHA6 | 16 | 1806692 | 1878608 | 1 | 2.04E+08 | 2.04E+08 |
| PELI2 | 10 | 68778347 | 68974093 | 14 | 56117814 | 56301526 |
| DAAM1 | 10 | 71730213 | 71916529 | 14 | 59188646 | 59371405 |
| NSD2 | 6 | 1.1E+08 | 1.1E+08 | 4 | 1871424 | 1982207 |
| SPSB4 | 1 | 1.29E+08 | 1.29E+08 | 3 | 1.41E+08 | 1.41E+08 |
| HABP4 | 8 | 84579583 | 84615685 | 9 | 96450201 | 96491336 |
| DIS3L2 | 2 | 1.2E+08 | 1.21E+08 | 2 | 2.32E+08 | 2.32E+08 |
| TBC1D22A | 5 | 1.18E+08 | 1.18E+08 | 22 | 46762617 | 47175699 |
| ADAM12 | 26 | 45848827 | 46238138 | 10 | 1.26E+08 | 1.26E+08 |
| INSR | 7 | 17280452 | 17403052 | 19 | 7112255 | 7294034 |
| SIK3 | 15 | 27942431 | 28060833 | 11 | 1.17E+08 | 1.17E+08 |
| CCND2 | 5 | 1.06E+08 | 1.06E+08 | 12 | 4273762 | 4405490 |
| GNA12 | 25 | 41099259 | 41171209 | 7 | 2728112 | 2844324 |
| NELFA | 6 | 1.1E+08 | 1.1E+08 | 4 | 1982714 | 2041903 |
| PAPPA2 | 16 | 59132781 | 59456250 | 1 | 1.76E+08 | 1.77E+08 |
| FNDC3B | 1 | 95891014 | 96173201 | 3 | 1.72E+08 | 1.72E+08 |
| IGF2BP3 | 4 | 32077891 | 32222388 | 7 | 23310209 | 23470467 |
| ADAMTS17 | 21 | 6514360 | 6924114 | 15 | 99971589 | 1E+08 |
| RAP1GAP2 | 19 | 24395974 | 24452796 | 17 | 2755705 | 3037739 |
| SYN3 | 5 | 71475847 | 71926718 | 22 | 32512552 | 33058372 |
| LAPTM4A | 11 | 78862495 | 78880461 | 2 | 20032650 | 20052028 |
| NCAPG | 6 | 38765969 | 38812051 | 4 | 17810902 | 17844862 |
| NHEJ1 | 2 | 1.08E+08 | 1.08E+08 | 2 | 2.19E+08 | 2.19E+08 |
| SPRED1 | 10 | 33950024 | 34069008 | 15 | 38252326 | 38357249 |
