# Supplementary material for: Using prior information from humans to prioritize genes and gene-associated variants for complex traits in livestock
Source: PLoS Genet. 2020 Sep 14;16(9):e1008780. doi: 10.1371/journal.pgen.1008780 (PMC7514049; doi:10.1371/journal.pgen.1008780)
Supplement: S5 Table — + Total number of markers in the genes divided by gene length (in 10kpb). 10kbps windows were used to avoid having values of less than 1 in the contingency table. *Cattle genes with 1 to 1 orthologs in humans (after QC); **Cattle genes within 10kb of the 164 lead SNPs from Bouwman et al. [12] that also have orthologs in humans; ***Human height genes prioritized as those that overlap with, or are within 10kkp either side of the 649 lead SNPs for height in Wood et al. [9], that also have orthologs in cattle. Fisher’s exact test of non-random association in gene marker density in cattle and humans (p-value = 0.87). (DOCX) [file pgen.1008780.s006.docx]

**S5 Table: Contingency table showing the overlap of cattle stature genes from Bouwman *et al*. [12] with human height genes from Wood *et al.* [9], with the average marker density of the genes in both species.**

|  | In Wood *et al*. [10] | | | Not in Wood *et al*. [10] | | | Marginal totals (rows) |
| --- | --- | --- | --- | --- | --- | --- | --- |
|  | Genes | Markers per gene^+^ in cattle | Markers per gene^+^ in humans | Genes | Markers per gene^+^ in cattle | Markers per gene^+^ in humans | Genes |
| In Bouwman *et al*. [12] | 10 | 96.3 | 7.2 | 67 | 93.8 | 9.3 | **77**** |
| Not In Bouwman *et al*. [12] | 358 | 92.2 | 9.6 | 13,307 | 96.1 | 8.4 | **13,665** |
| Marginal totals (columns) | **368***** |  |  | **13,374** |  |  | **13,742*** |

^+^ Total number of markers in the genes divided by gene length (in 10kpb). 10kbps windows were used to avoid having values of less than 1 in the contingency table. *Cattle genes with 1 to 1 orthologs in humans (after QC); **Cattle genes within 10kb of the 164 lead SNPs from Bouwman *et al*. [12] that also have orthologs in humans; ***Human height genes prioritized as those that overlap with, or are within 10kkp either side of the 649 lead SNPs for height in Wood *et al*. [9], that also have orthologs in cattle. Fisher’s exact test of non-random association in gene marker density in cattle and humans (p-value = 0.87).
