# Supplementary material for: Using prior information from humans to prioritize genes and gene-associated variants for complex traits in livestock
Source: PLoS Genet. 2020 Sep 14;16(9):e1008780. doi: 10.1371/journal.pgen.1008780 (PMC7514049; doi:10.1371/journal.pgen.1008780)
Supplement: S6 Table — Mantel-Haenszel chi-squared (corrected for gene length) = 35.6, p-value < 2.4e-9, common odds ratio = 4.1. (DOCX) [file pgen.1008780.s007.docx]

**S6 Table*:* Contingency table showing the overlap of cattle stature genes from Bouwman *et al*. [12] with human height genes from Yengo *et al.* [10], while accounting for gene size*.***

|  |  | Gene size | |
| --- | --- | --- | --- |
| In Bouwman *et al*. [12] | In Yengo *et al*. [10] | Short | Long |
| Yes | Yes | 7 | 23 |
|  | No | 25 | 22 |
| No | Yes | 736 | 787 |
|  | No | 8,850 | 3,293 |

Mantel-Haenszel chi-squared (corrected for gene length) = 35.6, p-value < 2.4e-9, common odds ratio = 4.1.
