# Supplementary material for: Using prior information from humans to prioritize genes and gene-associated variants for complex traits in livestock
Source: PLoS Genet. 2020 Sep 14;16(9):e1008780. doi: 10.1371/journal.pgen.1008780 (PMC7514049; doi:10.1371/journal.pgen.1008780)
Supplement: Attachment — Submitted filename: response to reviewer .docx [file pgen.1008780.s008.docx]

Reviewer #1: Minor points:

line 136 –“…. no significant (p<0.05) ….” The ‘p<0.05’ is redundant as the exact p value has been reported nearby. Please remove the (p<0.05).

AU: Thank you for the comment. The redundant ‘p<0.05’ has been removed from the revised manuscript, **line 136**.

line 386-390 – Please be specific about the coordinates of start and end of the gene. Assuming this includes beginning of 5’UTR till the end of 3’ UTR. If so, please include in the methods description.

AU: The gene coordinates were extracted from Ensembl and the coordinates are based on the longest canonical transcript of the gene. Thus, gene coordinates as presented in the manuscript include the beginning of the 5’ UTR until the end of the 3’ UTR of the longest canonical transcript of the gene. This has been made clear in the revised manuscript, **lines 389-391**:

“The start and end positions of a gene were defined as the beginning of the 5’ UTR and the end of the 3’ UTR of the gene, respectively.”

Figure2&3 Please make sure the y axis range is the same for both cattle and human graphs.

AU: The figures 2 & 3 have been re-made to have the same y-axis range.

Table S1. Is the density of markers in datasets used the same with regards the 10K window of association with lead SNPs? There is a chance that OR of orthologue genes on chips with different marker density and genome gaps, affect the total likelihood of associations with said phenotype. Adding the marker density of the 10Kbp associated window and its average per gene in both species could clarify this issue. If there is a significant density difference (could be assessed the same way by Fisher’s exact 2x1 table) per same orthologue gene, please include that in the result section lines 149-165. This point also lends itself to the gene size-stratification analysis that has already been done using Cochran-Mantel- Haenszel in the following section.

AU: Thank you for the comment. Please note that (imputed) sequence data were used for the GWAS in both species and not commercially available chips (Table 1). Therefore, the results presented in Tables S1 and S3 are not likely to be related to chip differences across species. Following your suggestion, we have compared the average marker density per 10kbp windows of association with lead SNPs from Bouwman et al. (2018), Wood et al. (2014) and Yengo et al. (2018) (top panels in S1 Fig.) with the average marker density per gene in each species (bottom panels in S1 Fig). We computed average marker density as total SNPs in a window or gene divided by the total length (in kbp) of the window or gene. In general, the distribution of marker density per window of association with lead SNPs is very similar to the distribution of marker density per gene in both species.

**S1 Fig**: Distribution of average marker density in the 10kbp windows of association with lead SNPs from Bouwman et al. [1], Wood et al. [2] and Yengo et al. [3], and the average marker density of genes in both cattle and humans. Average marker density were computed as total number of markers in a window or gene divided by the length (in kbp) of the window or gene.

Furthermore, to assess the impact of marker density on the results presented in table S1, we followed your suggestion and implemented Fisher’s exact test for the average marker densities in the genes tested (new TableS5, below). The test revealed that there is no evidence for non-random association in terms of marker density of genes in both species (p = 0.8651). An indirect but supporting evidence for this is the fact that gene LD scores (indirect measure of marker density), are not correlated across species (lines 131-146). Altogether, evidence indicate that the results in S1 table are robust to the effect of marker densities in both species.

We have included the following lines in the revised manuscript, **lines 163 -171**:

“To check if the average marker density in the 10kbp windows of association with lead SNPs is higher than the average marker density of genes in both species, we plotted the distribution of average marker density (total number of SNPs divided by length in kbp) of the 10kbp windows against the distribution of average marker density of genes in both species (S1 Fig). In general, we observed very similar distributions of average marker density between the windows of interest and the genes. Furthermore, the result of fisher’s exact test (S5 table) showed no evidence of non-random association in marker density of genes in cattle and humans (p = 0.87), indicating that the estimates of overlap of potential height genes in cattle and humans is less likely to be influenced by marker density”

We have also included the following clarifications in the materials and methods section of the revised manuscript, **lines 423-438**.

“Using the Biomart Ensembl tool, we identified the genes potentially affecting stature in cattle as those whose start or end position in the genome are located within 10kbp of the physical positions of the 163 lead SNPs for stature as reported in [12]. We identified 77 orthologous genes in total. Similarly, we identified a total of 368 human orthologous genes whose start or end positions in the genome are within 10kbp of the physical positions of the 649 lead SNPs for height as reported in [9]. We then checked the number of the 77 potential cattle genes that are part of the 368 potential human height genes.

Given the larger sample size used by [10], we wanted to know if there is a stronger overlap between the 77 cattle genes from [12], and human height genes from [10], as against those from [9]. To do this, we used the Biomart Ensembl tool to identify all human orthologous genes whose start or end positions in the genome are within 10kbp of the physical positions of the 3,290 near independent significant SNPs from [10], 1,553 genes in total. We then checked the number of the 77 potential cattle stature genes that are part of the 1,553 potential human height genes and tested if the number is more than can be expected by chance. In both cases, we used Fisher’s exact test”.

***S5 Table: Contingency table showing the overlap of cattle stature genes from [1] Bouwman et al. [1] with human height genes from Wood et al. [2], with the average marker density of the genes in both species.***

|  | In Wood *et al*. [2] | | | Not in Wood *et al*. [2] | | | Marginal totals (rows) |
| --- | --- | --- | --- | --- | --- | --- | --- |
|  | Genes | Markers per gene^+^ in cattle | Markers per gene^+^ in humans | Genes | Markers per gene^+^ in cattle | Markers per gene^+^ in humans | Genes |
| In Bouwman *et al*. [1] | 10 | 96.3 | 7.2 | 67 | 93.8 | 9.3 | **77**** |
| Not In Bouwman *et al*. [1] | 358 | 92.2 | 9.6 | 13,307 | 96.1 | 8.4 | **13,665** |
| Marginal totals (columns) | **368***** |  |  | **13,374** |  |  | **13,742*** |

^+^ Total number of markers in the genes divided by gene length (in 10kpb). 10kbps windows were used to avoid having values of less than 1 in the contingency table. *Cattle genes with 1 to 1 orthologs in humans (after QC); **Cattle genes within 10kb of the 164 lead SNPs from Bouwman *et al*. [1] that also have orthologs in humans; ***Human height genes prioritized as those that overlap with, or are within 10kkp either side of the 649 lead SNPs for height in Wood *et al*. [2], that also have orthologs in cattle. Fisher’s exact test of non-random association in gene marker density in cattle and humans (p-value = 0.87).
